# Intimate Relationship Between Stress and Human Alpha‑Herpes Virus 1 (HSV‑1) Reactivation from Latency

**DOI:** 10.1007/s40588-023-00202-9

**Published:** 2023-07-27

**Authors:** Clinton Jones

**Affiliations:** 1 College of Veterinary Medicine, Department of Veterinary Pathobiology, Oklahoma State University, Stillwater, OK 74078, USA

**Keywords:** HSV-1, Life-long latent infections, Reactivation from latency, Recurrent disease, Stress, Glucocorticoid receptor (GR), Krüppel-like factors

## Abstract

**Purpose of Review:**

Numerous studies concluded stress (acute, episodic acute, or chronic) increases the incidence of human alpha-herpes virus 1 (HSV-1) reactivation from latency in neurons. This review will summarize how stress stimulates viral gene expression, replication, and reactivation from latency.

**Recent Findings:**

Stress (capital S) stress-mediated activation of the glucocorticoid receptor (GR) accelerates reactivation from latency, whereas a corticosteroid-specific antagonist impairs viral replication and reactivation from latency. GR and specific stress-induced cellular transcription factors also stimulate viral promoters that drive expression of key viral transcriptional regulators: infected cell protein 0 (ICP0), ICP4, ICP27 and viral tegument protein (VP16). Hence, GR is predicted to initially stimulate viral gene expression. GR-mediated immune-inhibitory functions are also predicted to enhance viral replication and viral spread.

**Summary:**

Identifying cellular factors and viral regulatory proteins that trigger reactivation from latency in neurons may provide new therapeutic strategies designed to reduce the incidence of reactivation from latency.

## Introduction

### Latency‑Reactivation Cycle Is Essential for Recurrent Disease

HSV-1 infection of oral, ocular, or nasal cavities leads to life-long latent infections in neurons within trigeminal ganglia (TG), brainstem, and other parts of the CNS, reviewed in [1, 2]. The latency-reactivation cycle is customarily divided into 3 stages: establishment, maintenance, and reactivation. The hallmark of establishing and maintaining latency is lytic cycle viral gene expression is silenced, infectious virus is undetectable, and neurons survive infection. During maintenance of latency, viral DNA is organized as chromatin, which does not support high levels of lytic cycle viral gene expression [3], and the viral genome is circularized. In contrast to productive infection, the latency-associated transcript (LAT) is the only viral transcript abundantly expressed during latency. LAT is a complex locus that expresses several micro-RNAs, a stable long non-coding RNA, and 2 novel small non-coding RNAs, reviewed in [1, 2]. LAT gene products inhibit apoptosis [4–8] and expression of viral genes important for productive infection [8–10]. Hence, LAT promotes neuronal survival and sustains a pool of latently infected neurons that can reactivate from latency multiple times in a mouse model of infection [11].

Approximately 400,000 individuals in the USA suffer from HSV-1 ocular disease. Recurrent eye disease, for example, herpetic stromal keratitis [12, 13], causes tissue destruction and occasionally blindness [14]. Acyclovir treatment only reduces recurrent ocular disease by 41% [14] because most cases are due to reactivation from latency [15]. HSV-induced encephalitis (HSE) is the most common cause of sporadic and fatal encephalitis [16, 17]. Although HSE generally occurs in the temporal and frontal lobes, HSE can also occur in the brainstem [18, 19]. The majority of HSE cases are due to reactivation from latency [16, 17].

### Stressful Stimuli Correlate with Increased Episodes of Reactivation from Latency

Stress (acute, episodic acute, or chronic), fever, UV light, and heat stress increase the incidence of reactivation from latency in humans [20–22]. Surprisingly, these divergent stimuli activate the glucocorticoid receptor (GR). For example, stress increases cortisol, which activates GR via a liganded mechanism [23]. Furthermore, an inhibitor of cortisol production impairs heat-shock induced HSV-1 reactivation suggesting heat stress increases cortisol levels [24]. Thirdly, UV light induces GR phosphorylation and transcriptional activation via ligand-independent mechanisms [25, 26]. UVB and UVC light, but not UVA, increase cortisol production in human skin cultures, and UVB light increases corticosteroid production in C57BL/6 J mice [27, 28]. Finally, UV light triggers expression of certain enzymes regulated by GR activation. In summary, these different reactivation stressors share common signaling proteins, including GR activation.

**Fig. 1:**
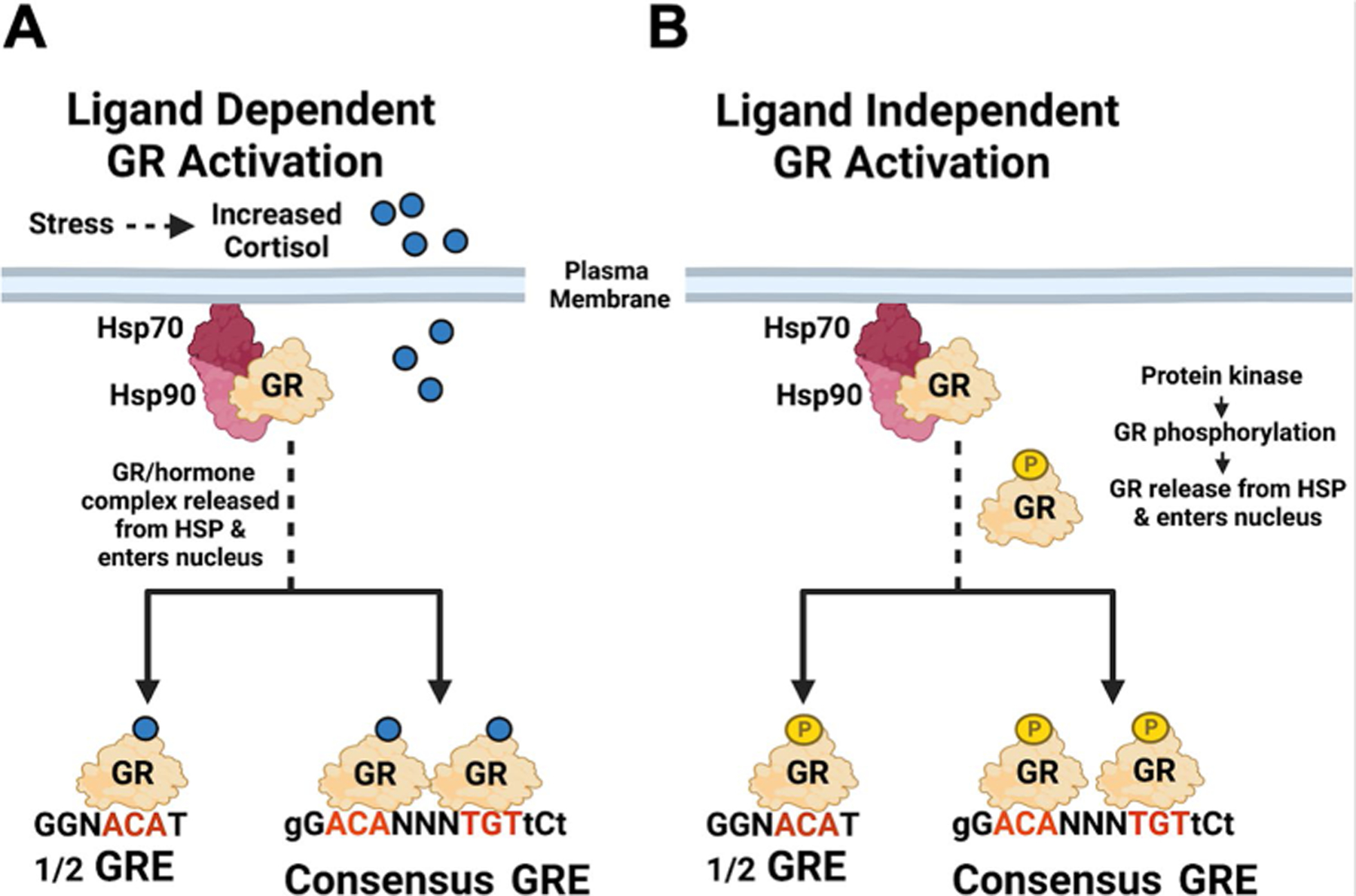
Activation Of Gr By Corticosteroids And Protein Kinases. **A** Schematic of key events that lead to GR activation by increased glucocorticoids secreted via the HPA. Red nucleotides in the GRE are essential nucleotides, capital letters are well conserved nucleotides, small letters are flexible, and N can be any nucleotide. A GR-hormone dimer specifically binds to a consensus GRE. A GR-hormone homodimer can also bind to a 1/2 GRE and stimulate transcription. **B** Certain protein kinases described in the text can phosphorylate GR, which promotes release of GR from the HSP complex (phosphorylated GR is denoted as GR-P). A phosphorylated GR dimer or GR monomer enters the nucleus, binds a consensus GRE or 1/2 GRE respectively, and transactivates promoters containing a 1/2 GRE. BioRender was used to generate this figure

The stress response is primarily mediated by secretion of glucocorticoids, including cortisol, via the hypothalamic-pituitary adrenocortical (HPA) axis, reviewed in [23]. Cortisol diffuses across the plasma membrane and interacts with GR. The GR-hormone complex disengages from the heat shock protein (HSP) complex, and the GR-hormone complex enters the nucleus. A GR-hormone homodimer binds to a consensus GR response element (GRE), remodels chromatin, and stimulates transcription, [29, 30] (Fig. 1A; ligand-dependent activation). This process occurs within minutes and does not require de novo protein synthesis. A GR monomer can also stimulate transcription by binding certain 1/2 GREs [31, 32]. Notably, GR can also stimulate gene expression via an un-liganded mechanism [25] (Fig. 1B). For example, GR phosphorylation at serine 134 is important for ligand-independent GR activation, culminating in gene expression (25). Serine 134 is hyperphosphorylated following glucose starvation, oxidative stress, UV irradiation, and osmotic shock suggesting cellular stressors directly induce GR phosphorylation at serine 134. The GR can be phosphorylated by mitogen-associated protein kinases (MAPKs), cyclin-dependent kinases (CDKs), glycogen synthase kinase 3 beta (GSK3B), and likely additional protein kinases [25]. Although the mineralocorticoid receptor (MR) can also bind cortisol, we predict MR is not as important during reactivation because MR does not activate transcription as efficiently as GR [33]. Approximately 50% of TG sensory neurons express GR [34] suggesting GR activation has the potential to directly induce reactivation from latency by stimulating viral gene expression. For example, GR can function as a pioneer transcription factor in vivo by interacting with nucleosomal sites and recruiting Brg1, which culminates in remodeling nucleosomes [35]. The hallmark of a pioneer transcription factor is they bind silent chromatin, activate transcription, and cell reprogramming [36, 37].

Corticosteroids also have anti-inflammatory and immune-suppressive effects, in part because GR binds two transcription factors: activator protein 1 (AP-1) and nuclear factor kappa-light-chain-enhancer of activated B cells (NF-_K_B), reviewed in [38, 39]. The AP-1 transcription factor can be a homodimer or a heterodimer and is comprised of four sub-families of transcription factors: Jun (c-Jun, JunB, JunD), Fos (c-Fos, FosB, Fra1, Fra2), Maf (musculoaponeurotic fibrosarcoma; c-Maf, MafB, MafA, Mafg/f/k, Nrl), and ATF (activating transcription factors; ATF2, LRF1/ATF3, BATF, JDP1, JDP2) protein families. AP-1 transcription factors bind a consensus DNA sequence (TGA(G/C)TCA), and the most common heterodimer bound to the consensus site is c-Fos and c-Jun. AP-1 regulates numerous immune checkpoints, including T cell activation, expansion of T helper subsets, and co-stimulation of T-cell responses, reviewed in [40]. GR-mediated inhibition of AP-1 transcriptional activity occurs, in part because GR directly interacts with the c-Jun subunit of Ap-1 [38, 39].

The NF-κB/Rel family includes NF-κB1 (p50/p105), NF-κB2 (p52/p100), p65 (RelA), RelB, and c-Rel, reviewed in [39, 41, 42]. Most members of this family can homodimerize or form heterodimers with each other. The most common activated form of NF-κB is a heterodimer consisting of p50 or p52 subunit and p65. NF-κB is generally localized in the cytoplasm and is inactive because it is associated with regulatory proteins called inhibitors of κB (IκB). In the canonical pathway, tumor necrosis factor-α for example, IκB is phosphorylated and subsequently degraded: consequently, NF-κB rapidly enters the nucleus, binds to promoters containing the consensus binding site 5′-GGGRNYY YCC-3′ (R is a purine, Y is a pyrimidine, and N is any nucleotide), and activates expression of numerous genes that encode innate immune or pro-inflammatory regulators. GR inhibits NF-_K_B-dependent transcription by directly interacting with p60, recruiting histone deacetylases to NF-_K_B-dependent promoters, and/or preventing phosphorylation of the C-terminus of RNA Pol II [38, 39]. Finally, corticosteroids can readily induce apoptosis in certain lymphocyte subsets, which will reduce immune responses [38, 39]. In summary, increased corticosteroid levels are predicted to increase the incidence of reactivation by more than one mechanism.

### Viral Proteins Predicted to Mediate Early Stages of Reactivation from Latency

When TG cultures obtained from mice latently infected with HSV-1 are infected with an adenovirus vector that expresses ICP0, ICP4, or virion protein 16 (VP16), reactivation from latency is induced [43]. These viral proteins are key viral transcriptional regulators and possess functions required to initiate lytic cycle viral gene expression during reactivation from latency. For example, ICP0 is a relatively large protein,110 kDa, that stimulates immediate early (IE), early (E), and late (L) HSV-1 gene expression (44); disrupts nuclear domain 10 structures [45]; and evades host intrinsic and innate antiviral defenses [46, 47]. Furthermore, ICP0 possesses E3 ubiquitin ligase activity that is crucial for its functions, reviewed in [48]. The ICP4 protein, a 175 kDa phosphoprotein, specifically binds multiple sites on the viral genome [49] where it recruits the TATA box-binding protein and RNA pol II transcription factor IIB to activate early and late viral gene expression (50). Consequently, ICP4 is essential for productive infection [51], and its expression triggers production of infectious virus during reactivation from latency. ICP0 and ICP4 mRNA are expressed as IE genes during productive infection; hence, these viral proteins are readily detected early after infection. The viral tegument protein (VP16) is expressed as a leaky-late protein that interacts with two cellular proteins: host cellular factor 1 (HCF-1) and Oct-1. This multi-protein complex binds specific sequences in IE promoters and transactivates all IE promoters, reviewed in [52–54]. Thus, expression of ICP0, ICP4, or VP16 could initiate lytic cycle viral gene expression and virus production during reactivation from latency.

### HSV‑1 Models for Studying the Latency‑Reactivation Cycle

An in vivo heat-stress model of reactivation from latency in TG neurons of mice concluded VP16 is essential for reactivation [55, 56]. A rat primary superior sympathetic neuronal model of latency also concluded VP16 initially drives reactivation from latency [57, 58]. This model predicts two phases drive production of infectious viruses [58]. The hallmark of the first phase includes de-repression of silent lytic viral genes, and this phase does not require viral proteins. The hallmarks of the second phase include nuclear localization of VP16 and host cell factor 1 (HCF-1), an essential VP16 transcriptional coactivator. These two phases precede increased viral gene expression and production of infectious virus. This same rat model reported ICP0 expression occurs after VP16 because ICP0 expression overcomes interferon treatment [59]. Additional rodent neuronal cell models of latency have provided insight into certain aspects of the HSV-1 latency-reactivation cycle, reviewed in [60]. Human embryonic neuronal precursor cells, Lund human mesencephalic (LUHMES), proliferate when expression of a tetracycline-regulatable (Tet-off) v-myc transgene is induced, and these cells can support HSV-1 latency [61]. LUHMES can be readily differentiated into neuronal-like cells, and the virulent HSV-1 strain (17syn +) reactivates more efficiently than a less virulent strain (KOS) [62] indicating cell culture model of latency is a useful model to compare to results from rodent neuronal models. Many of the cell culture models of latency require treatment of the antiviral drug, acyclovir, to stop viral gene expression and establish a quiescent infection.

When TG from mice latently infected with HSV-1 are dissected, minced into smaller pieces, and then placed in media, virus shedding consistently occurs, and this procedure is referred to as explant-induced reactivation. During explant-induced reactivation, LAT gene products are reduced [63], HCF-1 is rapidly recruited to IE promoters [64], chromatin remodeling of the ICP0 promoter occurs, ICP0 transcription occurs [65], and infectious virus is produced. The synthetic corticosteroid dexamethasone (DEX) accelerates explant-induced reactivation [66, 67], and a GR-specific antagonist, CORT-108297, impairs reactivation [67] indicating GR activation is important for this process. Immunohistochemistry studies revealed VP16 is detected prior to ICP0 and ICP4 during DEX-induced reactivation from latency [67]. Assuming these antibodies are equally effective for detecting viral proteins in formalin-fixed and paraffin-embedded thin sections, this finding appears to support the concept that VP16 is expressed early during reactivation from latency. An independent study using TG explants concluded that viral RNA expression is disordered during explant-induced reactivation [63].

Rabbits latently infected with the McKrae strain, a neurovirulent HSV-1 strain, undergo spontaneous reactivation from latency [68] or reactivation induced by iontophoresis [69]. UV light also triggers reactivation from latency in mice latently infected with HSV-1 [70]. Notably, DEX treatment of calves or rabbits latently infected with bovine alphaherpesvirus 1 (BoHV-1) is the only α-herpesvirus member where reactivation from latency is reproducibly initiated [71, 72]. Cell culture models of latency and explant-induced reactivation are important: however, they may not recapitulate all complex virus-host interactions that occur during in vivo reactivation.

### Identification of Stress‑Induced Cellular Transcription Factors

Using transcriptomic approaches, stress-induced cellular transcription factors were identified in TG when calves latently infected with BoHV-1 are treated with DEX, which consistently initiates rapid reactivation from latency [73]. Expression of Krüppel like factor 4 (KLF4), KLF6, KLF15, promyelocytic leukemia zinc finger (PLZF), Slug (also referred to as Snail homolog 2), and Sam-pointed domain containing Ets transcription factor (SPDEF) [73] was significantly increased when calves latently infected with BoHV-1 were treated with DEX for 3 h to initiate reactivation from latency. Interestingly, KLF15, Slug, and SPDEF are also expressed in more mouse TG neurons following explant when treated with DEX confirming these cellular transcription factors are part of the stress response [74]. In response to stress, GR and KLF15 regulate gene expression dynamics via a feed-forward loop [75, 76]. The hallmark of this feed-forward loop is GR stimulates KLF15 expression and GR and KLF15 form a stable complex and activate expression of genes in specific pathways, including enhanced expression of amino acid metabolizing enzymes and adipogenesis [75, 76].

### GR and Stress‑Induced Cellular Transcription Factors Activate ICP0, ICP4, and VP16 Promoter/Regulatory Sequences

**Fig. 2:**
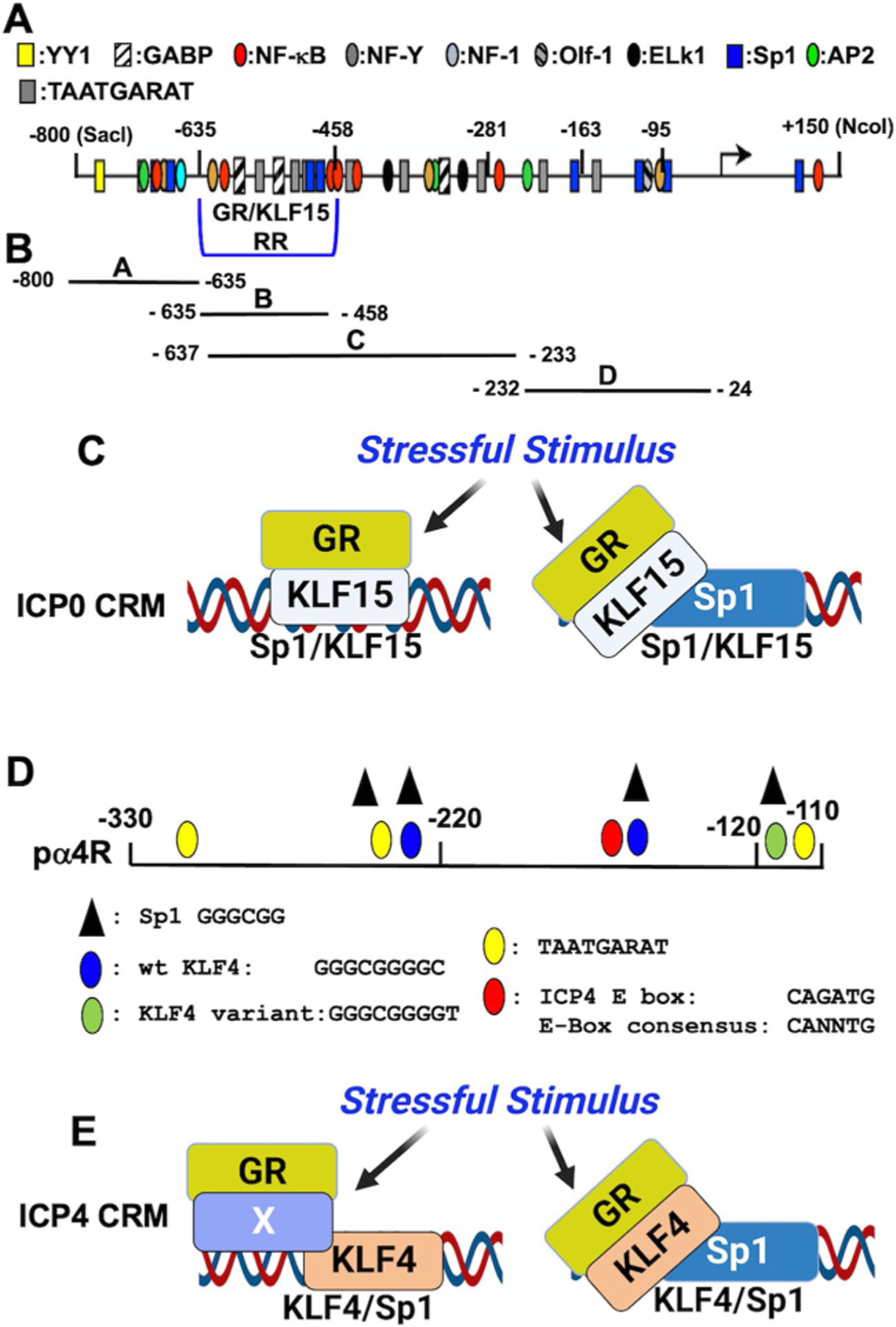
Summary of HSV-1 IE promoter/regulatory sequences and how they are transactivated by (GR) and stress-induced transcription factors. **A** Schematic of ICP0 promoter and location of GR/KLF15 responsive region (GR/KLF15 RR), transcription factor binding sites, and 1/2 GREs. **B** Four fragments (A–D) derived from ICP0 promoter sequences upstream of the TATA box were inserted upstream of a minimal promoter upstream that drives firefly luciferase expression. **C** Putative model depicting two potential scenarios demonstrating how GR and KLF15 or GR, KLF15, and Sp1 transactivate ICP0 CRMs. **D** Schematic of wt ICP4 CRM (pα4R) and consensus binding sites of the denoted transcription factors. Nucleotide numbers (− 330 and − 110) are relative to the ICP4 transcription initiation site. **E** Putative model depicting two possible scenarios describing how GR and KLF15 or GR, KLF15, and Sp1 transactivate ICP0 CRMs. BioRender was used to generate **C** and **E** in this figure

The ability of GR and/or stress-induced transcription factors to transactivate promoter/regulatory sequences of the ICP0, ICP4, or VP16 genes was examined in transient transfection studies. The rational for these studies is that ectopic expression of these genes initiates reactivation from latency in TG cultures prepared from latently-infected mice [43]. An ICP0 promoter fragment spanning − 800 to + 150 relative to the transcription initiation site was initially examined (Fig. 2A) because this construct is stimulated by heat stress [77]. GR, KLF15, and DEX treatment cooperatively transactivate the full-length ICP0 promoter in spite of no consensus GREs [78]. Conversely, the other stress-induced transcription factors discussed in “Identification of Stress-Induced Cellular Transcription Factors” section did not have a profound effect. Four cis-regulatory modules (CRMs) that span ICP0 promoter sequences upstream of the TATA box were inserted at the 5’ terminus of a simple promoter that drives luciferase activity (Fig. 2B). All but the CRM C fragment was cooperatively transactivated by GR, KLF15, and DEX in Vero cells. Conversely, GR or KLF15 and DEX are sufficient for transactivation in Neuro-2A cells (Fig. 2B) [79]. Mutagenesis of Sp1 binding sites (GGGCGG or CCG CCC) in fragments A, B, and D reduced transactivation by GR, KLF15, and/or DEX to basal levels. GR and KLF15 occupy ICP0 promoter sequences in transfected cells and early times after infection [78] supporting the concept that these transcription factors directly regulate ICP0 promoter activity (Fig. 2C). These studies do not preclude the possibility that Sp1 recruits the KLF15/GR complex to a Sp1 binding site (Fig. 2C), or other transcriptional cofactors mediate this process.

An ICP4 CRM inserted upstream of a minimal promoter in a luciferase reporter vector (Fig. 2D; denoted as pα4R) is cooperatively transactivated by GR, DEX, and PLZF, Slug, KLF15, or KLF4 in Neuro-2A or Vero cells [80]. Two KLF4 binding sites and a variant KLF4 binding sites are in ICP4 CRM sequences (Fig. 2D). These KLF4 binding sites contain a consensus Sp1 binding site. GR-, DEX-, and KLF4-, KLF15-, or PLZF-mediated transactivation is reduced to basal transcriptional levels when the two consensus KLF4 binding sites are mutated [80]. Interestingly, an enhancer box (E-Box), which Slug is known to bind to [81, 82], and the adjacent 3’ KLF4 binding site are essential for GR-, DEX-, and Slug-mediated transactivation. Notably, the 3’ KLF4 binding, but not the KLF4 site adjacent to the E-box, is important for GR, DEX, and KLF4, KLF15, or PLZF cooperative transactivation. Like GR, KLF4 is a pioneer transcription factor [36, 37]. Hence, we propose binding of GR and KLF4 with KLF4 consensus binding sites (Fig. 2E) may be particularly important for activating ICP4 expression following stressful stimuli. Sp1 and/or unknown transcriptional coactivators may also play a role in GR, KLF4, and DEX cooperative transactivation following stress (Fig. 2E; denoted by X).

**Fig. 3:**
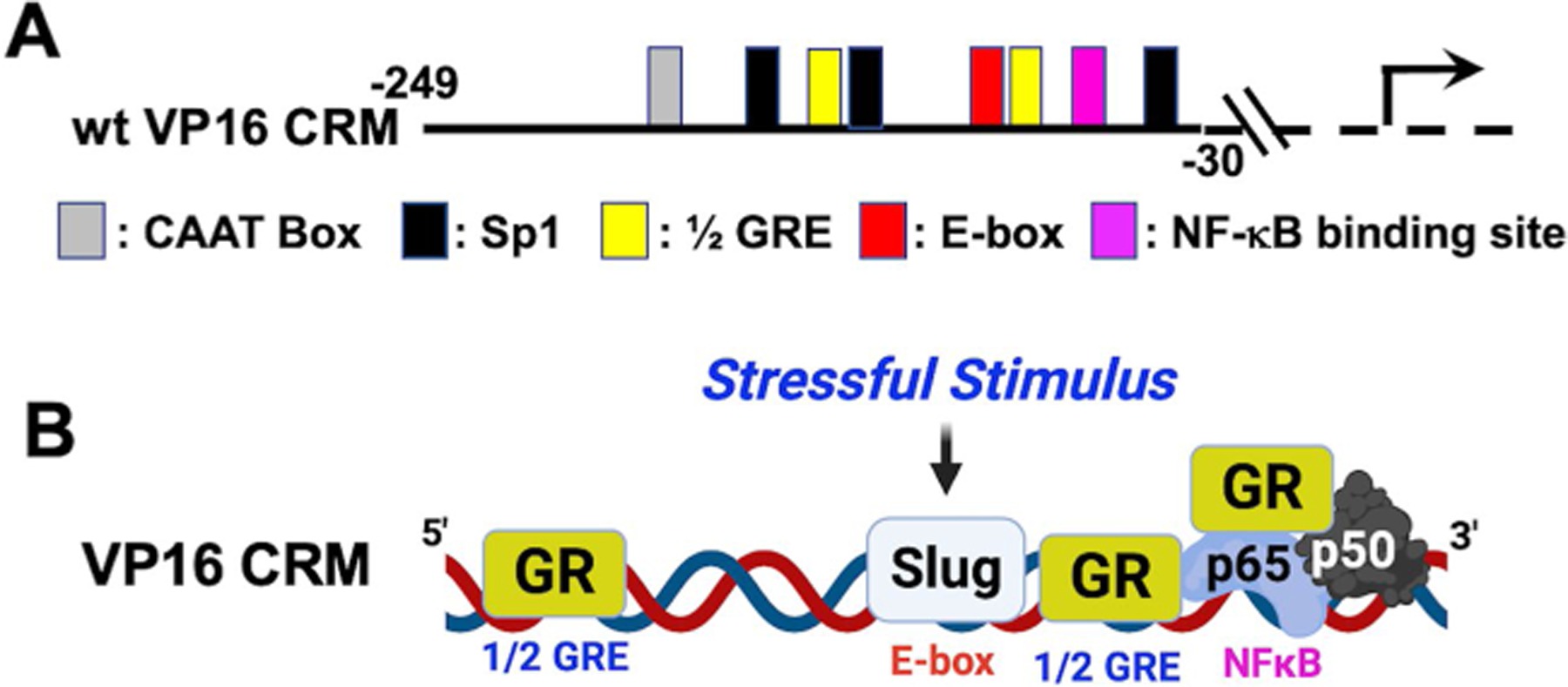
Schematic of VP16 CRM and how GR and Slug can transactivate these sequences. **A** Schematic of the wt VP16 CRM construct and location of transcription factor binding sites. Nucleotide numbers (− 249 and − 30) are relative to the VP16 transcription initiation site. **B** Putative model summarizing how GR and Slug transactivate the VP16 CRM. BioRender was used to generate **B** in this figure

A recent study revealed that GR and Slug transactivate a VP16 CRM (Fig. 3A) in an additive fashion, and when Slug is silenced, productive infection is impaired [82]. DEX has no effect on GR- and Slug-mediated transactivation. In contrast to ICP0 and ICP4 CRMs, GR and stress-induced KLF family members (KLF4, KLF15, or PZLF) do not transactivate the VP16 CRM in mouse fibroblasts (NIH3T3 cells) or Neuro-2A cells (Fig. 3A) [82]. Furthermore, mutating all 3 Sp1 binding sites had no effect on GR- and Slug-mediated transactivation. The consensus E-box, 1/2 GREs, or NF-_K_B binding site were crucial for GR- and/or Slug-mediated transactivation (Fig. 3B). Although Slug was originally defined as a transcriptional repressor, Slug can activate transcription by binding E-boxes upstream of certain promoters [81, 82].

### Identification of Cellular Factors that Mediate HSV‑1 Reactivation in Mouse Models of Infection

Various transgenic mouse strains are available that could provide insight into which cellular genes influence the HSV-1 latency-reactivation cycle. For example, a recent study tested whether HCF-1 plays a role in the latency-reactivation cycle. HCF-1 interacts with Oct-1 and VP16 to initiate IE promoters during productive infection [64]. This complex also promotes formation of active histone modifications, but impairs accumulation of repressive histone markers on IE promoters, reviewed in [83]. HCF-1 nuclear localization correlates with reactivation indicating this protein mediates reactivation from latency [84]. As expected, primary fibroblasts from HCF-1 knockout mice do not support efficient viral replication relative to primary fibroblasts from wild-type mice [85]. To test whether HCF-1 influences reactivation from latency, a mouse strain containing a 5’loxP site at Exon 2 with a selectable marker and 3’ loxP site at Exon 3 of HCF-1 was used to specifically knockout HCF-1 expression in TG neurons [85]. HSV-1 recombinant viruses expressing the Cre recombinase under control of the HSV ICP0 or LAT promoter were used to delete exon 2 and 3 of HCF-1 during acute infection of TG neurons. Explant-induced reactivation was significantly reduced in HCF-1 conditional knockout mice relative to wt C57Bl/6 mice when infected with ICP0 or LAT expressing CRE viruses. Utilizing an independent approach to knock out HCF-1 in TG neurons, reactivation from latency was also impaired confirming HCF-1 plays an important role during reactivation from latency.

A mouse strain where the murine GR contains a serine 229 to alanine mutation (GR^S229A^) was compared to wt mice to examine the effect of GR transcriptional activity on viral replication during acute infection, establishment of latency, and explant-induced reactivation from latency [86]. The mouse GR serine 229, and its human homologue located at GR serine 211, must be phosphorylated for optimal GR-mediated transcriptional activation, reviewed in [38]. Mutating serine 211 of the human GR also induces conformational changes in the GR activation function region 1, which correlates with reduced transactivation of promoters containing GREs [39]. These studies revealed that explant-induced reactivation from latency is impaired in female, but not male GR^S229A^ mice. As expected, wt HSV-1 McKrae strain induced the same levels of explant-induced reactivation in parental wt C57Bl/6 mice regardless of sex. Furthermore, HSV-1 replication in primary kidney fibroblasts prepared from GR^S229A^ mice (females or males) was reduced relative to primary kidney fibroblasts prepared from parental wt mice. The reason why GR has a female-specific effect during explant-induced reactivation is currently not understood.

## Conclusions

While there is controversy about which viral protein initiates reactivation from latency, it is reasonable to predict ICP0, ICP4, or VP16 can be a lead player in triggering reactivation from latency in different neurons. For example, there are four known subtypes of TG neurons based on cell surface markers, and HSV-1 establishes latency in distinct populations of TG neurons versus HSV-2 [87]. Furthermore, HSV-1 establishes latency in neurons in brainstem [88], autonomic ciliary ganglia [89], and other neurons in the central nervous system. As discussed above, stress, fever, UV light, and heat stress increase the incidence of reactivation from latency in humans, and GR is induced by these cellular stressors via liganded or unliganded mechanisms. Hence, cellular stressors, neuron-specific stress-induced transcription factors, and cellular signaling pathways are likely to dictate whether ICP0, ICP4, or VP16 is initially expressed during reactivation from latency.

During reactivation from latency, GR activation is predicted to play important roles by initially activating viral gene expression. The ability of GR to suppress immune responses and inflammation [41] is predicted to enhance viral spread to peripheral cells and tissue. GR, the progesterone receptor, and androgen receptor belong to the same family of nuclear hormones and can bind GREs [90, 91] suggesting these receptors and their cognate hormones influence reactivation from latency in certain neurons and via sex-dependent mechanisms.

Although mammals, including humans, are exposed to daily stressors, HSV-1 does not reactivate from latency every day. Cellular factors and LAT-encoded gene products are predicted to actively maintain a latent infection. For example, immune-mediated processes will likely impair virus shedding and spread during reactivation from latency. Recent studies revealed serine-threonine protein kinases (Akt1 and Ak2) interfere with stress-induced transcription [92]. While Akt3 did not impair stress-induced transcription, it promotes neurite formation when Neuro-2A cells are differentiated by reducing serum levels in the media. Akt activation is enhanced by the phosphoinositide 3-kinase signaling cascade [93], which is proposed to promote maintenance of latency in certain models of latency, reviewed in [60]. The Wnt/β-catenin signaling pathway also maintains Akt activation and is activated during HSV-1 and BoHV-1 latency [94, 95]. LAT gene products are predicted to impair expression of key lytic cycle viral genes and productive infection [1, 2, 8–10] suggesting these function promote establishment and/or maintenance of latency. Conversely, stress-induced transcription factors that are pioneer transcription factors, GR [35] and KLF4 [37], for example, are predicted to play a crucial role during early stages of reactivation from latency because viral promoters that drive expression of key transcriptional regulators exist as silent chromatin during latency [3].
